# Elevated lactate in Mauriac syndrome: still a mystery

**DOI:** 10.1186/s12902-021-00835-1

**Published:** 2021-08-21

**Authors:** Brice Touilloux, Henri Lu, Belinda Campos-Xavier, Andrea Superti-Furga, Michael Hauschild, Thérèse Bouthors, Christel Tran

**Affiliations:** 1 Center for Molecular Diseases, Division of Genetic Medicine, Lausanne University Hospital, University of Lausanne, Lausanne, Switzerland; 2 Respiratory Medicine Department, Lausanne University Hospital, University of Lausanne, Lausanne, Switzerland; 3 Service of Cardiology, Department of Cardio-Vascular Medicine and Surgery, Lausanne University Hospital, University of Lausanne, Lausanne, Switzerland; 4 Pediatric Endocrinology, Diabetology and Obesity Unit, Lausanne University Hospital, University of Lausanne, Lausanne, Switzerland

**Keywords:** Mauriac syndrome, Glycogenic hepatopathy, Elevated lactate

## Abstract

**Background:**

The Mauriac syndrome was described in 1930 as a peculiar combination of poorly controlled diabetes mellitus type 1, stunted growth and glycogenic hepatopathy. More recently, lactic acidosis was recognized as an additional feature, often induced by insulin treatment.

**Case presentation:**

A 17-year old girl known for diabetes type 1A and Mauriac syndrome was admitted to the emergency room with hyperglycemia of > 41 mmol/l without ketoacidosis. Under a standard insulin regimen, hyperglycemia was rapidly corrected but marked hyperlactatemia occurred.

**Conclusions:**

The mechanism of impaired glucose utilization and lactate elevation independent of ketoacidosis in Mauriac syndrome is intriguing. The rarity of Mauriac syndrome and its resemblance to glycogen storage diseases suggest the presence of a specific metabolic or genetic predisposition that remains to be identified.

## Background

In 1930, P. Mauriac described children who had diabetes mellitus type 1, enlarged liver with glycogen accumulation, poor growth and delayed pubertal development [1]. Accumulation of glycogen within the hepatocytes leading to liver enlargement and/or elevation of liver enzymes is defined as glycogenic hepatopathy; it may occur also in adults with poorly controlled type 1 diabetes [2]. Glycogenic hepatopathy can be associated with lactic acidosis, particularly following insulin treatment [3, 4]. In a series of reported children with Mauriac syndrome, approximately half had elevated lactate levels despite no signs of illness or diabetic ketoacidosis (DKA) [5]. In DKA, elevation of lactate may be associated to glycolysis induced by hyperglycemia and seems to vary according to ketogenesis [6, 7]. Thus, there seems to be a metabolic abnormality in Mauriac syndrome that differs from, or occurs in addition to, insulinopenic diabetes. The pathophysiological mechanisms and hypothetical causes responsible for this constellation are still the subject of discussion.

## Case presentation

**Fig. 1:**
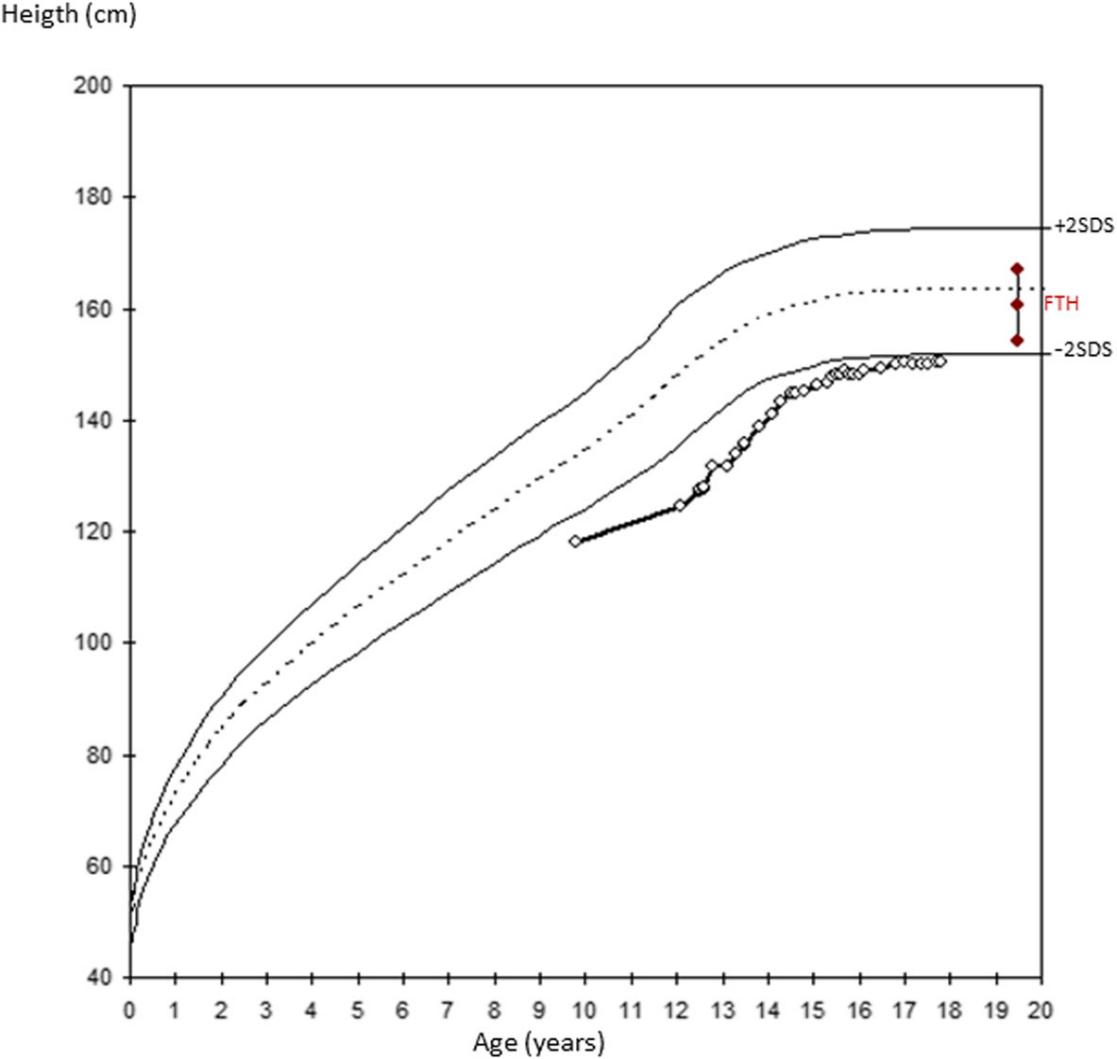
Growth chart of the reported patient showing height from the age of 9 years. Familial target height was calculated based on the formula = ((Height of Father + Height of Mother) / 2) - 6.5 cm [9]. Length < 3rd Percentile based on the WHO (World Health Organization) growth charts for girls [10]. FTH: Familial Target Height

A 17-year-old girl, was admitted to the adult emergency room (ER) with a 4 days history of mild abdominal pain and nausea. She was well known in pediatric endocrinology for diabetes type 1A (positive anti-glutamic acid decarboxylase and anti-insulin antibodies). The past medical history was notable for diabetes type 1A diagnosed in Ecuador at age 4 years. Access to insulin therapy had been limited until the age of 9, resulting in poorly controlled diabetes. Following her migration to Switzerland at age 12, she was noted to have protuberant abdomen, severe hepatomegaly (17.5 cm to the midclavicular line, uniform echogenicity on abdominal ultrasound), growth retardation, Cushingoid features, moon face and proximal muscle wasting leading to the clinical diagnosis of Mauriac syndrome. IGFBP-3 was borderline low 2.5 and 3.5 mg/l (reference values for 12 years 2.9–8.6) possibly related with growth impairment [8]. Initial optimization of insulin therapy allowed for better diabetes control, normalization of liver size and some catch-up growth; however, her height remained under familial target (Fig. 1). Treatment included long-acting insulin at a dose of 0.21 UI/kg/day and short acting insulin at a mean dose of 0.43 UI/kg/day. Unfortunately, non-compliance recurred, and diabetes remained undercontrolled with glycated hemoglobin increasing progressively from 8, 0% to 13, 5% despite close follow-up. Liver size remained normal.

**Fig. 2:**
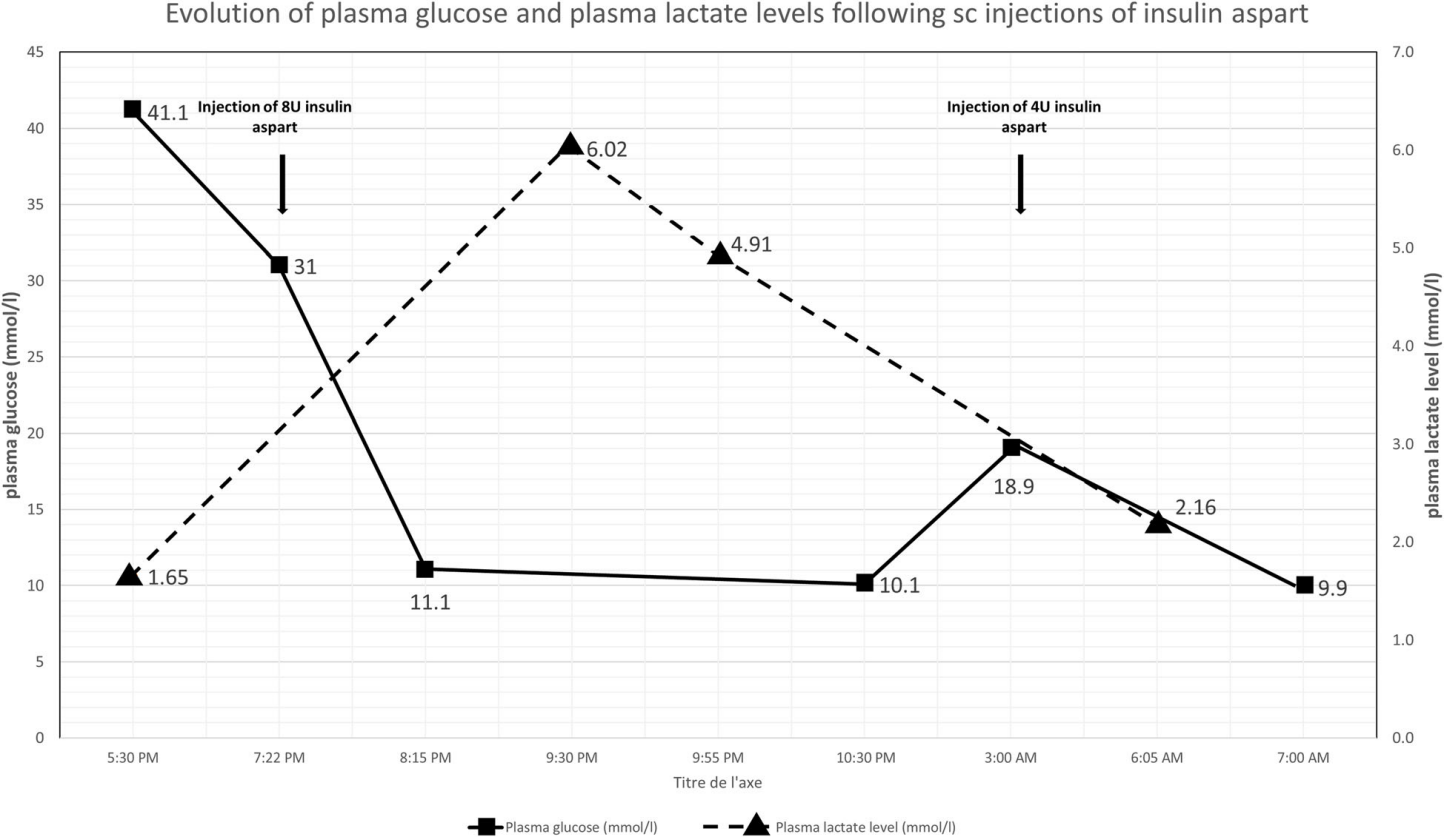
Evolution of plasma glucose (mmol/l) and plasma lactate (mmol/l) levels following injections of subcutaneous insulin aspart. SC: Subcutaneous

Upon admission to the ER at age 17, she was afebrile with heart rate of 87 beats/min and blood pressure of 122/99 mmHg. Her height was 149.3 cm (< 3rd percentile; WHO growth curves [10]) and her weight was 59.5 kg (P50-P75) with a body mass index of 26.69 kg/m^2^. Tanner stage was 5 (post-pubertal). Abdominal examination revealed mild tenderness on palpation but no rebound and no hepatomegaly. Her blood glucose level was 44.1 mmol/l without acidosis (pH – 7.376, pCO _2_–39.8 mm of Hg, HCO _3_–22.9 meq/l, base excess – 2.4 mmol/l), and urine ketones were 1.5 mmol/l (normal, < 0.3 mmol/l). There was mild hyperosmolar hyponatremia (Na - 130 mmol/l, osm − 316 mmol/kg H2O). Liver transaminases, lactate, blood cell count, kidney function tests were normal. Her HbA1c was 11%. Acute hyperglycemia with no signs of ketoacidosis was recognized as the cause of abdominal pain and nausea. Management included fluid replacement as a rate of 20 ml/kg/h for 2 h which lead to a fall in serum glucose from 41.1 mmol/l to 31 mmol/l. Subsequently, rapid acting subcutaneous insulin therapy at a dose of 0.13 U/kg (8 Unit) was given. When serum glucose reached 11.1 mmol/l, fluid replacement was stopped. A second insulin injection at a dose of 0.07 U/kg (4 Unit) was given 7 h later. During infusion and insulin therapy, lactate levels increased from 1.65 mmol/l prior to insulin administration to 6.02 mmol/l 125 min after the injection of insulin aspart (8 U, Fig. 2). Blood pH remained normal at 7.367 (Table 1). Following the next 12 h, lactate levels progressively decreased to 2.16 mmol/l (Fig.1). Urine ketones were repeated at this time with similar result as the day of admission (1.5 mmol/l). While we did not determine plasma ketones directly, the absence of acidosis and the low urinary ketones at admission and the day after, as well as the absence of clinical signs of DKA indicate that hyperlactatemia did not develop secondary to DKA. Abdominal ultrasonography and magnetic resonance imaging showed a normal liver parenchyma and liver size (15 cm to the midclavicular line).

**Table 1:** Blood gasometric values, sodium chloride 0.9% and insulin aspart injection (sc) during hospital admission

	5:30 pm (Day 1)	7:22 pm	8:15 pm	9:30 pm	9:55 pm	10:30 pm	3:00 am (Day 2)	6:05 am	7:00 am
Arterial blood gas									
- pH	7.376			7.367	7.387			7.421	
- HCO _3_ (meq/l)	22.9			19.2	20.6			23.8	
- base excess (mmol/l)	−2.4			−6.2	−4.3			−1.0	
- anion gap	9.1			14.8	13.8			9.2	
SC injection of insulin aspart (U)		8					4		
Sodium chloride 0.9% intravenous infusion (cc/h)	1000 Total: 1800 cc infused	500 Total: 300 cc infused	Stop			83	83	83	Stop

*U* Unit; *SC* subcutaneous

As genetic variants at the *KJCN11* and *PHKG2* genes had been previously described in association with Mauriac syndrome [11, 12], an exome gene panel for glycogen storage diseases including *AGL, GYS2, PHKA1, PHKA2, PHKB, PHKG2, PYGL* and *KCNJ11* was analyzed with next generation sequencing technology. No pathogenic variants were identified.

## Discussion and conclusions

The key findings in this case are hyperglycemia in the absence of ketoacidosis and elevated lactate following insulin administration. This constellation has been observed in other individuals with the Mauriac syndrome and remains poorly understood [3, 4]. In view of its rarity, it seems unlikely that Mauriac syndrome be simply the result of poor diabetic control. Given that glycogenic hepatopathy, hyperlactatemia and hypoglycemia are hallmarks of the liver genetic glycogen storage diseases, a defect in glycogen metabolism has been suggested in Mauriac syndrome. In normal conditions, glucose entering the hepatocyte is phosphorylated to glucose 6-phosphate and can follow two main metabolic pathways, namely, glycogen synthesis and glycolysis. In glycogenic hepatopathy, chronic hepatic glycogen overload might impair the incorporation of available intracellular glucose to “new” glycogen and thus divert glucose to glycolysis. Pyruvate, the end product of glycolysis is converted to lactate when the Krebs cycle is saturated [4]. Correction of blood glucose by insulin would further divert glucose carbons into lactate, due to inhibition of glucose-6-phophatase [13]. The observation of an elevated lactate/pyruvate ratio in a patient with Mauriac syndrome is of interest, although, as the authors mention, other rare genetic causes such as a respiratory chain defect associated with primary mitochondrial diabetes should be kept in mind [2], especially when diabetes-related autoantibodies are negative. Furthermore, the patient had no risk factors for thiamine deficiency (i.e. unbalanced diet, recurrent vomiting, or previous gastrointestinal surgery) that can cause unexplained elevated lactate, given that thiamine is a cofactor for the enzyme pyruvate dehydrogenase [14].

Is glycogenic hepatopathy the key to the pathogenesis of Mauriac syndrome? MacDonald et al. found a heterozygous pathogenic variant in the *PHKG2* gene encoding for liver glycogen phosphorylase kinase (PhK), a regulatory protein kinase responsible for glycogen breakdown, in a patient with Mauriac syndrome [12]. However the mother was also carrier of this variant and asymptomatic. Tomihira et al. sequenced the gene *PYGL* coding for the liver phosphorylase enzyme in a patient with diabetes type 1 and hepatomegaly but did not identify pathogenic variants [15]. In addition, no pathogenic variant in the *PHKG2* gene or in related genes was identified in our patient. Thus, the contribution of genetic defects in glycogen metabolism leading to glycogenic hepatopathy remains to be clarified.

The clinical and metabolic constellation of Mauriac syndrome are typically seen in children, and changes tend to normalize in adulthood with resolution of hepatomegaly, cytolysis and growth failure except for diabetes control. However rare observations like ours suggest that the peculiar metabolic predisposition to hyperlactataemia when treating hyperglycemia with insulin may persist in adulthood [2, 4]. Monitoring of plasma lactate levels during insulin administration for hyperglycemia may be indicated in Mauriac syndrome. What remains unclear is whether there is a specific constitutional predisposition (genetic or acquired) that leads to the metabolic constellation of Mauriac syndrome, and what this predisposition might be. For the future, studies of glycogen metabolism in Mauriac syndrome using non-invasive techniques coupled with a thorough genetic analysis of genes involved in glucose regulation might be revealing.

## Data Availability

The data of this study may be available on reasonable request to the corresponding author.

## Abbreviations

DKA: Diabetic ketoacidosis
ER: Emergency room
WHO: World health organization
