# Correction to: Elevated lactate in Mauriac syndrome: still a mystery

**DOI:** 10.1186/s12902-021-00858-8

**Published:** 2021-09-30

**Authors:** Brice Touilloux, Henri Lu, Belinda Campos-Xavier, Andrea Superti-Furga, Michael Hauschild, Thérèse Bouthors, Christel Tran

**Affiliations:** 1 Center for Molecular Diseases, Division of Genetic Medicine, Lausanne University Hospital, University of Lausanne, Lausanne, Switzerland; 2 Respiratory Medicine Department, Lausanne University Hospital, University of Lausanne, Lausanne, Switzerland; 3 Service of Cardiology, Department of Cardio-Vascular Medicine and Surgery, Lausanne University Hospital, University of Lausanne, Lausanne, Switzerland; 4 Pediatric Endocrinology, Diabetology and Obesity Unit, Lausanne University Hospital, University of Lausanne, Lausanne, Switzerland

**Correction to: BMC Endocr Disord 21, 172 (2021)**

**https://doi.org/10.1186/s12902-021-00835-1**

Following publication of the original article [1], two errors were found in the article:

1. The affiliation of the co-author Therese Bouthors should be: Pediatric Endocrinology, Diabetology and Obesity Unit, Lausanne University Hospital, University of Lausanne, Lausanne, Switzerland.
2. On page 2, the figure citation in the sentence ‘Following the next 12 h, lactate levels progressively decreased to 2.16 mmol/l (Fig. 1)’ is incorrect, it should be ‘Following the next 12 h, lactate levels progressively decreased to 2.16 mmol/l (Fig. 2)’.

The original paper has been updated.
